# Skin-Contact Triboelectric Nanogenerator for Energy Harvesting and Motion Sensing: Principles, Challenges, and Perspectives

**DOI:** 10.3390/bios13090872

**Published:** 2023-09-06

**Authors:** Ali Matin Nazar, Reza Mohsenian, Arash Rayegani, Mohammadamin Shadfar, Pengcheng Jiao

**Affiliations:** 1 Donghai Laboratory, Zhoushan 316021, China; matin.22@intl.zju.edu.cn; 2 Zhejiang University-University of Illinois at Urbana-Champaign Institute, Zhejiang University, Haining 314400, China; 3 College of Health and Rehabilitation Sciences, Sargent College, Boston University, Boston, MA 02215, USA; rm2002@bu.edu; 4 Centre for Infrastructure Engineering, Western Sydney University, Kingswood, NSW 2747, Australia; a.rayegani@westernsydney.edu.au; 5 School of Medicine, Zhejiang University, 866 Yuhangtang Rd., Hangzhou 310058, China; amin.shadfar@zju.edu.cn; 6 Institute of Port, Coastal and Offshore Engineering, Ocean College, Zhejiang University, Zhoushan 316021, China

**Keywords:** self-powered sensors, energy harvesting, triboelectric nanogenerators (TENG), skin-contact TENG, structural health monitoring

## Abstract

Energy harvesting has become an increasingly important field of research as the demand for portable and wearable devices continues to grow. Skin-contact triboelectric nanogenerator (TENG) technology has emerged as a promising solution for energy harvesting and motion sensing. This review paper provides a detailed overview of skin-contact TENG technology, covering its principles, challenges, and perspectives. The introduction begins by defining skin-contact TENG and explaining the importance of energy harvesting and motion sensing. The principles of skin-contact TENG are explored, including the triboelectric effect and the materials used for energy harvesting. The working mechanism of skin-contact TENG is also discussed. This study then moves onto the applications of skin-contact TENG, focusing on energy harvesting for wearable devices and motion sensing for healthcare monitoring. Furthermore, the integration of skin-contact TENG technology with other technologies is discussed to highlight its versatility. The challenges in skin-contact TENG technology are then highlighted, which include sensitivity to environmental factors, such as humidity and temperature, biocompatibility and safety concerns, and durability and reliability issues. This section of the paper provides a comprehensive evaluation of the technological limitations that must be considered when designing skin-contact TENGs. In the Perspectives and Future Directions section, this review paper highlights various advancements in materials and design, as well as the potential for commercialization. Additionally, the potential impact of skin-contact TENG technology on the energy and healthcare industries is discussed.

## 1. Introduction

As technology continues to evolve, there is an increasing demand for portable and wearable devices that can operate without the need for frequent battery replacements [1,2,3]. In response to this demand, energy-harvesting technologies have gained significant attention in recent years [4]. Skin-contact triboelectric nanogenerator (TENG) technology has emerged as a promising solution for energy harvesting and motion sensing, offering an efficient way to convert mechanical energy from human motions into electrical energy. However, this technology can also be applied in other fields, for example, utilizing it as a smart sensor in structural smart control systems [5,6]. In this review paper, we provide a detailed overview of skin-contact TENG technology, covering its principles, challenges, and perspectives for energy-harvesting and motion-sensing applications [7,8,9,10,11,12,13]. This study begins by defining skin-contact TENG and discussing the importance of energy harvesting and motion sensing [14]. The principles of skin-contact TENG are based on the triboelectric effect. When two materials come into contact and separate, the contact electrification generates energy [15,16]. The surface properties of materials, including their roughness and chemical composition, exert a significant influence on the magnitude of energy generated in triboelectric nanogenerator (TENG) systems. Material roughness impacts the effective contact area between the triboelectric layers, directly affecting the charge transfer during frictional interactions. A rougher surface increases the actual contact points, promoting more efficient charge separation and transfer, and thus enhancing energy output. Additionally, the chemical nature of the material’s surface can affect its electron affinity, which plays a critical role in the triboelectric series and subsequent charge polarization. Materials with dissimilar electron affinities tend to experience more pronounced charge redistribution during contact, resulting in stronger triboelectric effects and higher energy conversion efficiency. Consequently, a comprehensive understanding and manipulation of these surface properties offers opportunities to optimize TENG performance by tailoring material selection and surface engineering strategies [17,18]. The working mechanism of skin-contact TENG is based on the relative sliding motion between two surfaces, which results in a charge transfer and the production of an electric potential difference. The materials used in skin-contact TENG are typically flexible and lightweight, as they need to be able to conform to the surface of the skin. Various materials including silicone, PDMS, fabrics, and metals have been used to construct TENGs [19].

Skin-contact TENG technology has numerous potential applications in fields ranging from wearables to healthcare [20]. Energy harvesting for wearable devices is the most prominent application of skin-contact TENGs [20]. For example, they can be integrated into smartwatches, fitness trackers, and other wearables that monitor physical activity. Moreover, skin-contact TENGs can harvest energy from various body motions, such as pressure, vibration, shear, and torsion. Motion sensing is another significant application of skin-contact TENGs [21,22,23,24]. In healthcare monitoring, skin-contact TENGs can be used to detect muscle or joint movement, respiration rate, heartbeat, and pulse, which makes them valuable tools in healthcare and wellness industries [25]. In addition to wearables and healthcare, the integration of skin-contact TENGs with other technologies, such as wireless communication or energy storage systems, has potential for further applications. Despite the potential benefits of skin-contact TENG technology, several challenges need to be addressed to ensure its full potential [26]. The sensitivity of skin-contact TENGs to environmental factors, such as humidity, temperature, and mechanical vibrations, can limit the efficiency of energy harvesting. Biocompatibility and safety concerns associated with the materials used must also be considered [27,28,29,30,31]. Other challenges include the durability and reliability of skin-contact TENGs when subjected to repeated movements and stresses. Perspectives on and future directions of skin-contact TENG technology depend on advancements in materials and design [32]. Advancements in materials could lead to more efficient TENGs with a high output power density and better biocompatibility. Improved design and optimization of TENGs could lead to reduced sensitivity to environmental factors and increased durability and reliability [33,34,35,36,37,38,39]. The potential for commercialization of skin-contact TENG technology is significant given the growing market for wearables and remote health monitoring [40].

### Fundamentals of Triboelectric Nanogenerators

**Figure 1:**
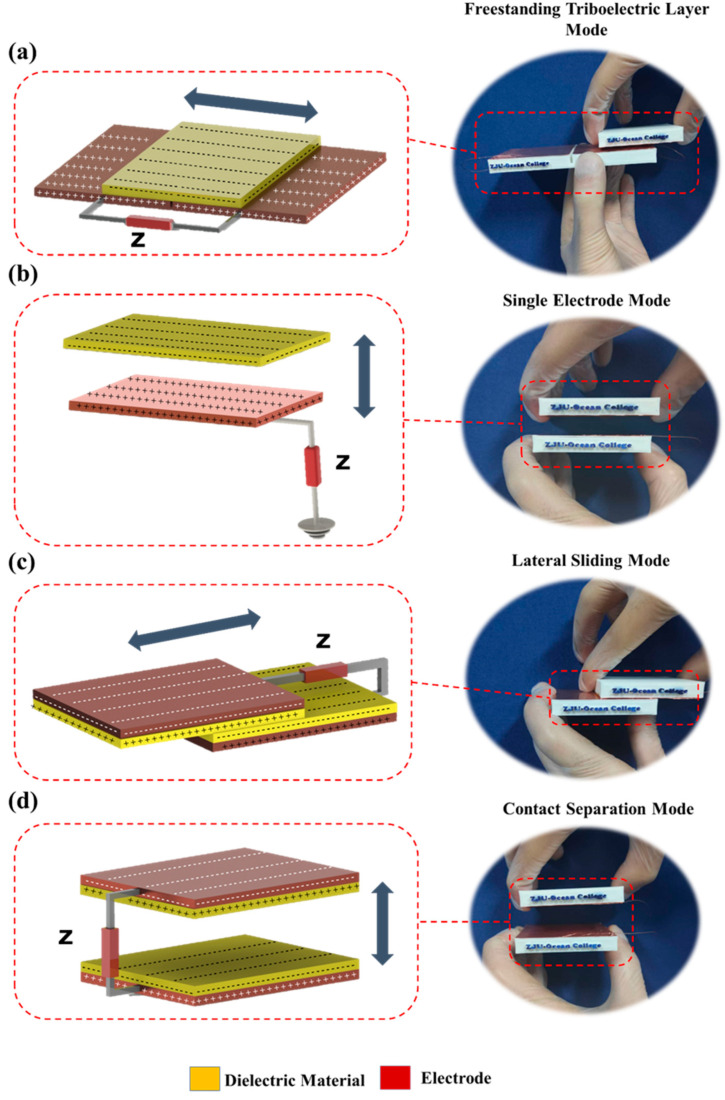
TENG modes: (contact separation mode, lateral sliding mode, single electrode mode, freestanding triboelectric layer mode) [17].

Figure 1 illustrates four distinct operational modes inherent to triboelectric nanogenerators (TENG), each characterized by its own unique attributes and merits: the freestanding triboelectric layer mode (depicted in Figure 1a), the single electrode mode (depicted in Figure 1b), the lateral sliding mode (depicted in Figure 1c), and the contact separation mode (depicted in Figure 1d). These TENG operational modes are designed to facilitate the transference of electrostatic charges to the connected electrodes. With the exception of the single electrode mode, all TENG modes necessitate the involvement of a pair of electrodes, as portrayed in Figure 1. Upon subjecting a TENG layer to movement, its initial electrostatic mode undergoes a transition, resulting in the emergence of a discernible potential difference. This potential disparity serves as the impetus behind the movement of charges, thereby effecting an eventual attainment of electrostatic equilibrium in the external circuit. Altering the alignment of the electrodes to face counter to the orientation of the TENG layer leads to an inversion in the observed potential difference. This intrinsic reciprocity in the motion engenders an alternating current (AC) output, characterizing the distinctive attribute of TENG. The contact separation mode entails the presence of dual electrodes positioned at the rear of the TENG layers. The act of contact and subsequent separation engenders a distinct potential difference, amenable to measurement. The quantification of output voltage can be achieved by introducing a voltmeter across the span of the two electrodes. Analogously, the lateral sliding mode also features a pair of electrodes positioned beneath the TENG layers. The relative displacement of these layers precipitates a departure from the electrostatic equilibrium, culminating in the creation of a discernible potential difference. The resulting voltage consequent to this potential gradient can be gauged by leveraging the reciprocal motion intrinsic to the TENG layers, thereby enabling measurement via a voltmeter connected to the dual electrodes. The provision of the Single Electrode Mode represents a strategy wherein a sole electrode interfaces with an external load, in lieu of the conventional pairing of two electrodes. In this configuration, when the TENG layer makes contact with the electrode, the electrostatic equilibrium is disrupted, inducing the flow of current. Contrasting this, the freestanding triboelectric layer mode involves independent movement of the TENG layer vis-à-vis the electrode. The orchestrated sliding of the TENG layer amidst the electrodes gives rise to a noticeable potential difference [17,41].

## 2. Principles of Skin-Contact Triboelectric Nanogenerators 

Skin-contact triboelectric nanogenerator (TENG) technology is an innovative solution for energy harvesting and motion sensing applications. TENG technology is based on the triboelectric effect, which is the generation of electricity due to the transfer of electrons between two surfaces that have different electronegativities [42,43,44,45,46,47,48,49]. Skin-contact TENGs utilize the triboelectric effect to convert the mechanical energy from human movements into electrical energy in order to harvest energy [50]. This paper will discuss the principles of skin-contact triboelectric nanogenerators, focusing on the triboelectric effect and its role in energy harvesting, the working mechanism of skin-contact TENG, and the materials used in skin-contact TENG [51,52,53,54,55,56,57,58,59]. The transfer of electrons causes an electrical potential difference to be created between the two surfaces [8,60]. The presence of an electrical potential difference between two points has the potential to be exploited for energy extraction. This can be achieved by converting mechanical energy, which is generated from motion or forces, into electrical energy. In essence, the electrical potential difference serves as a resource for generating electricity through the transformation of mechanical energy. Skin-contact TENGs use the triboelectric effect to generate electrical energy by converting the motion of the human body into electrical energy [8,9,61,62,63,64,65,66,67].

**Figure 2:**
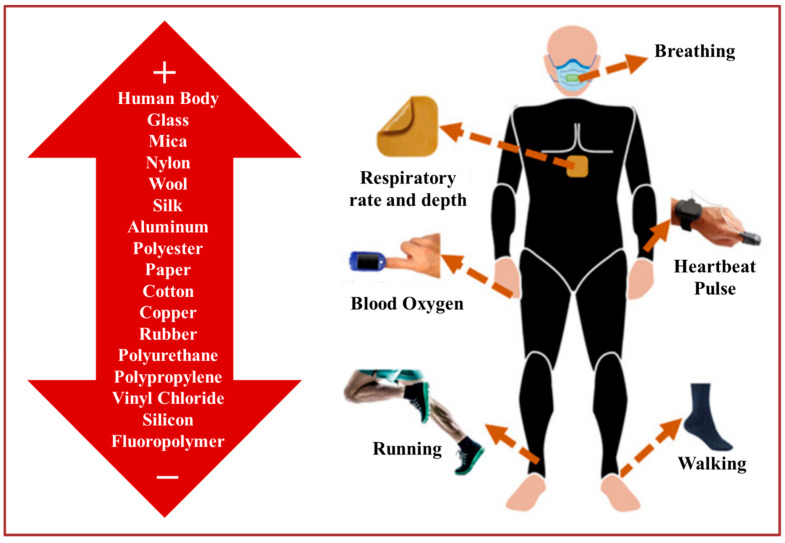
Principles of skin-contact triboelectric nanogenerator [68].

Figure 2 shows the working mechanism of skin-contact TENG is based on the relative sliding motion between two surfaces that come into contact [68,69]. The skin-contact triboelectric nanogenerator comprises two distinct electrodes positioned on opposite sides of a dielectric layer. This arrangement is pivotal, facilitating the TENG’s core functionality of converting mechanical energy from skin-induced interactions into electric energy, achieved by exploiting the triboelectric effect and generating an electric potential difference across the isolated electrodes. The electrodes and the dielectric layer are constructed from materials that have different electronegativity values [70,71,72,73]. This electric potential difference can be used to harvest the energy generated by the mechanical motion of the human body [74]. The materials used in skin-contact TENGs are typically flexible, lightweight, and biocompatible to ensure that they do not irritate the skin [25,75,76,77,78,79,80,81,82]. The dielectric layer can be made of materials such as silicone, polyethylene terephthalate (PET), and polydimethylsiloxane (PDMS) [83]. The electrodes can be made of various materials, including gold, silver, copper, and other conductive materials [84]. The selection of materials is an essential aspect of the TENG design as it affects the magnitude of the electrical energy generated by the TENG [85]. Generally, possible materials for triboelectric nanogenerator (TENG) applications include various combinations of triboelectric and conductive materials for effective charge generation and collection. Triboelectric materials often include polymer films like polydimethylsiloxane (PDMS), polyimide, and polyethylene, along with metals like aluminum, copper, gold, and silver. Flexible substrates such as polyimide and PET provide flexibility, while biocompatible substrates like medical-grade silicones ensure safe skin contact. Dielectric materials like polyethylene and polypropylene create potential differences, while conductive polymers like polyaniline and polypyrrole serve as effective electrodes. Additional functional coatings like hydrophobic, biocompatible, and antimicrobial coatings can enhance TENG performance and safety. In specialized applications, piezoelectric materials and flexible electronics components might also be integrated for motion sensing and structural health monitoring.

## 3. Applications of Skin-Contact Triboelectric Nanogenerator

The skin-contact triboelectric nanogenerator (TENG) is a promising technology that has various applications in different fields. One of the most significant applications of TENG is energy harvesting for wearable devices [86]. With the increasing demand for portable and efficient energy sources, TENGs can generate electricity from human body movements and provide power to wearable devices such as smartwatches, fitness trackers, and medical devices [87]. Additionally, TENGs can be used for motion sensing in healthcare monitoring, where they can detect and analyze body movements, vital signs, and physical activities [88]. Moreover, TENGs can be integrated with other technologies such as piezoelectric nanogenerators (PENG) to develop more advanced and sophisticated systems for various applications [89,90,91,92].

### 3.1. Energy Harvesting for Wearable Devices

**Figure 3:**
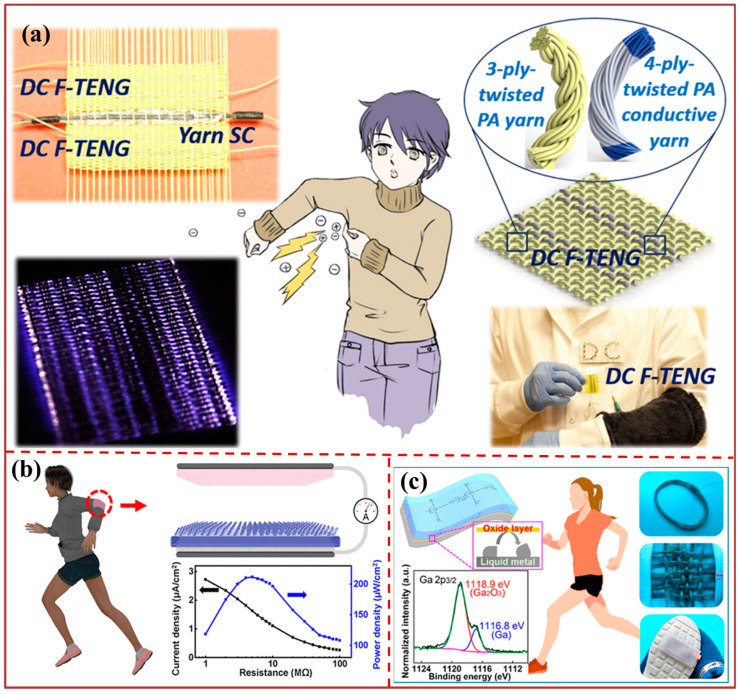
Energy harvesting for wearable devices: (**a**) Working mechanism of the DC F-TENG [93]. (**b**) Structural design of a WTNG to harvest mechanical energy from human motions [94]. (**c**) Structure design of the bracelet-like LM-TEMG for harvesting mechanical energy from arm shaking [95].

The energy harvesting for wearable devices based on triboelectric nanogenerators is depicted in Figure 3. Figure 3a illustrates a direct current (DC) TENG fabric (DC F-TENG) with the most prevalent plain structure, which is designed to harvest biomotion energy by deftly exploiting the harmful and irritating electrostatic breakdown phenomenon of clothing. 

The DC F-TENG device, which is both cost-effective and efficient, has the potential to serve as a wearable energy-harvesting solution in the future. It can generate DC energy directly, without the need for a rectifier bridge, by harnessing energy from electrostatic breakdown. Additionally, its lightweight and flexible design make it a comfortable option for users. The utilization of an oblique microrod array in the construction of a textile-based wearable triboelectric nanogenerator (WTNG) is demonstrated in Figure 3b, representing a novel structure that exhibits high performance. The effective enlargement of the contact area in WTNGs can be achieved through the uniform bending and unidirectional sliding of oblique poly(dimethylsiloxane) microrods under operational circumstances. The present study proposes a proficient methodology to improve the output efficacy of triboelectric nanogenerators, thereby establishing a propitious pathway to energizing wearable electronic devices. 

**Figure 4:**
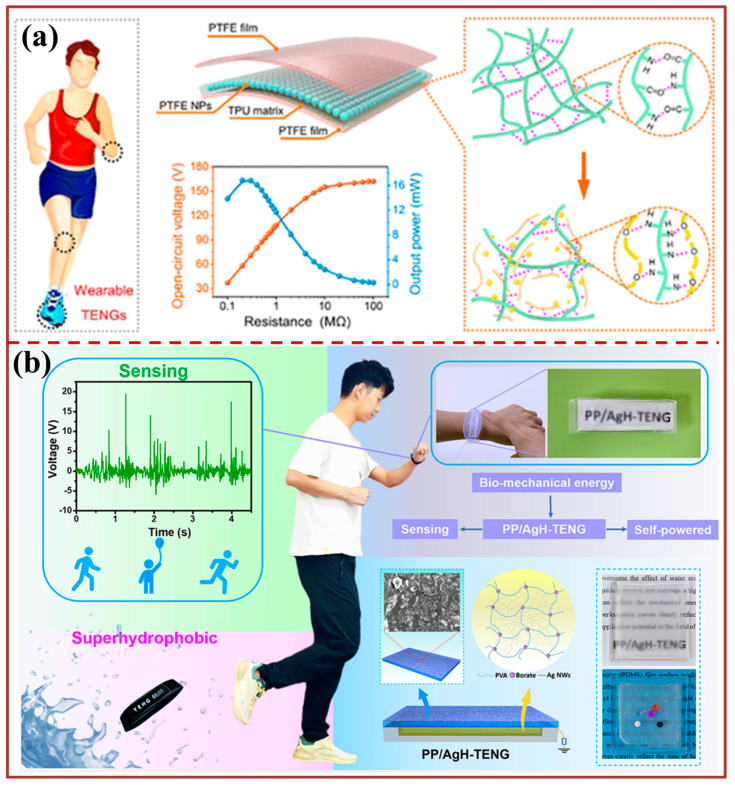
Energy harvesting for wearable devices: (**a**) Demonstrations of the TENG utilized to harvest biomechanical energy from different parts of the human body [96]. (**b**) Application of PP/AgH-TENG in self-powered sensing and energy harvesting [97].

The LM-TENG utilizing Galinstan as the electrode and silicone rubber as the triboelectric and encapsulation layer is presented in Figure 3c. The liquid metal’s Young’s modulus, being relatively small, guarantees the electrode’s uninterrupted conductivity even when subjected to deformations, stretching up to a strain of approximately 300%. The remarkable stability of the Galinstan device is attributed to the surface oxide layer that effectively inhibits the further oxidation and permeation of the liquid Galinstan electrode into the silicone rubber. Moreover, the LM-TENG exhibits consistent operational efficiency when subjected to diverse deformations, including stretching, folding, and twisting. Various forms of LM-TENGs, including bulk-shaped, bracelet-like, and textile-like, have the capability to extract mechanical energy from human motion, such as walking, arm shaking, or hand patting, in order to power wearable electronic devices in a sustainable manner. The proposed optimization strategy for materials, as depicted in Figure 4a, aims to attain a superior performance of triboelectric nanogenerators (TENGs) while concurrently reducing the matching impedance. The triboelectric layer in question is a composite film consisting of a thermoplastic polyurethane (TPU) matrix, polyethylene glycol (PEG) additives, and polytetrafluoroethylene (PTFE) nanoparticle inclusions. This film is capable of tuning permittivity through electret properties. By means of optimizing the dielectric constant of the composite material, the injected charge density and internal capacitance of the triboelectric nanogenerator (TENG) are substantially increased, resulting in a synergistic enhancement of the output power and a reduction in the TENG’s impedance. The present study showcases a noteworthy advancement in the enhancement of materials for a triboelectric generator, thereby facilitating its practical implementation in commercial settings. The study showcases the preparation of a core-shell superhydrophobic and flexible triboelectric nanogenerator, denoted as PP/AgH-TENG, as depicted in Figure 4b. The device was fabricated using a polydimethylsiloxane (PDMS) film that underwent surface modification with polytetrafluoroethylene (PTFE) particles, serving as the triboelectric layer. Additionally, AgNWs/PVA hydrogel was utilized as the electrode. The ability of PP/AgH-TENG to effectively mitigate the impact of water molecules on charge transfer in a moist atmosphere while expeditiously recuperating and sustaining a substantial output is of significant importance. Furthermore, the wristband composed of PP/AgH-TENG has the capability to accumulate the mechanical energy produced by human motion, and the resultant output performance graphs distinctly depict the condition of human activity. The present study exhibits significant potential for practical implementation in the domain of intelligent wearable self-powered sensing.

### 3.2. Motion Sensing for Healthcare Monitoring

**Figure 5:**
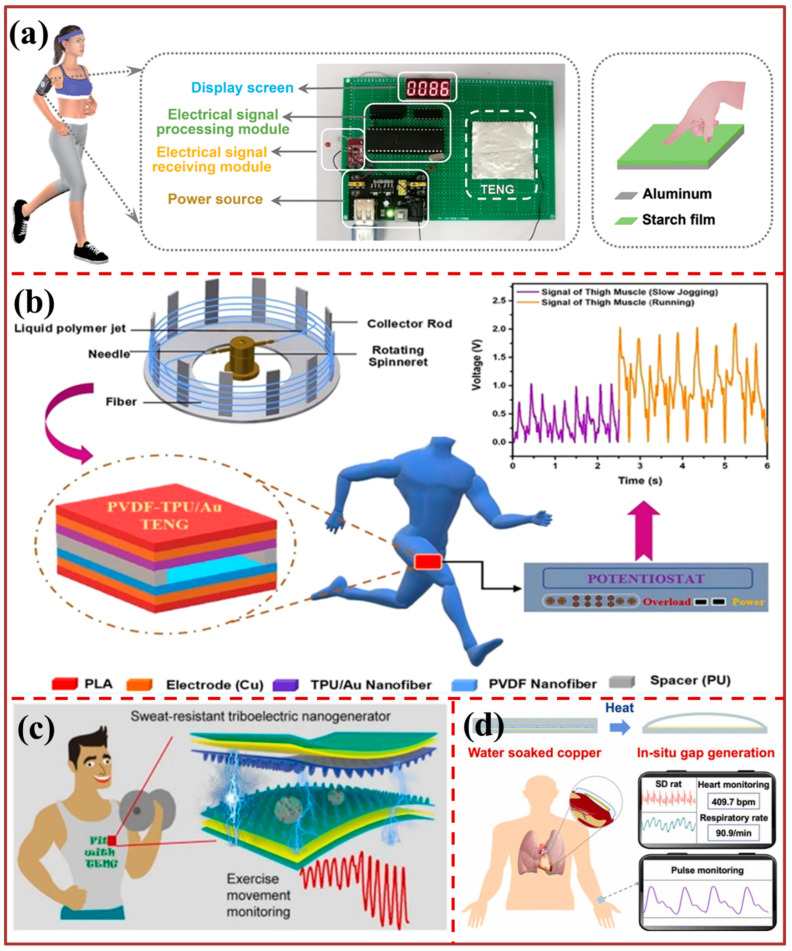
Motion sensing for healthcare monitoring: (**a**) Structure design of starch triboelectric nanogenerator (S-TENG) for behavior monitoring [98]. (**b**) Sensing performance of PTA-TENG for different body motions [99]. (**c**) Schematic structure of the BSRW-TENG [100]. (**d**) Schematic illustration showing the basic structure of the NSTENG [77].

**Figure 6:**
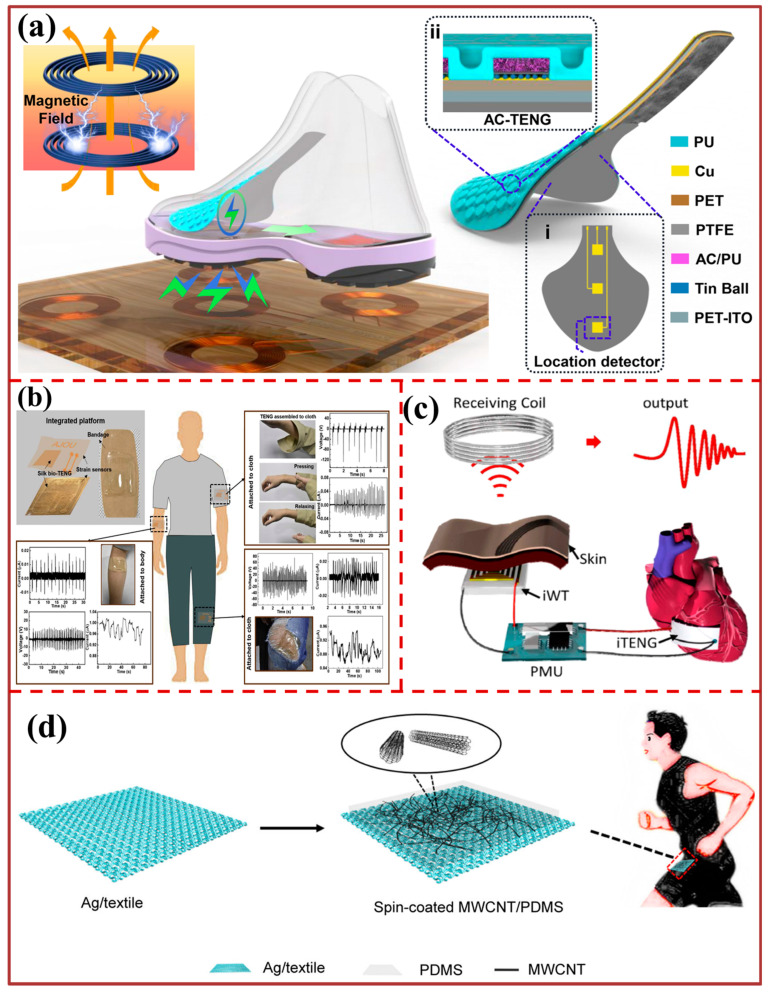
Motion sensing for healthcare monitoring: (**a**) Design principal of AC-TENG [101]. (**b**) Schematic diagram and photograph images of the integrated strain sensor and the bio-TENG on a bandage [87]. (**c**) Device structure, surface modification, and cytocompatibility of the iTENG [78]. (**d**) Structure design and application of single-electrode triboelectric nanogenerator (S-TENG) [102].

Figure 5 and Figure 6 showcase the utilization of motion sensing technology across various healthcare monitoring levels. In Figure 5a, the diagram illustrates the creation of a versatile and eco-friendly wheat starch triboelectric nanogenerator (S-TENG) using a simple and sustainable approach. The S-TENG demonstrates an open-circuit voltage of 151.4 V and a short-circuit current of 47.1 μA. This technology not only facilitates the operation and intelligent regulation of electronic devices, but it also efficiently harnesses energy from bodily motions and wind. Notably, the S-TENG’s performance remains unaffected by increased ambient humidity levels; instead, it exhibits an unexpected enhancement. In the humidity range of 20% RH to 80% RH, the S-TENG device exhibits significant potential as a highly sensitive self-powered humidity sensor. Furthermore, the S-TENG technology streamlines the large-scale production of biomaterial-based TENG, enabling the creation of self-powered sensing and wearable devices with multifunctional capabilities. Figure 5b illustrates the application of an all-nanofiber-based TENG for energy harvesting and biomechanical sensing. The TENG utilizes poly(vinylidene fluoride) (PVDF) and thermoplastic polyurethane (TPU) nanofiber membranes, produced via the Forcespinning (FS) method. Integration of TPU nanofiber membranes with a uniformly deposited gold nanofilm, achieved through sputtering, is demonstrated.

The findings of the experimental investigation on the PVDF-TPU/Au NF-TENG indicate that the surface of the TPU fiber membrane interfaced with dispersed gold resulted in a maximum open-circuit voltage of 254 V and a short-circuit current of 86 μA output at a 240 bpm load frequency. These values were observed to be 112% and 87% higher, respectively, than those obtained from the bare PVDF-TPU NF-based TENG. The study presents the findings of a bioinspired sweat-resistant wearable triboelectric nanogenerator (BSRW-TENG) designed for monitoring physical activity during exercise, as depicted in Figure 5c. The BSRW-TENG is composed of a dual-layered triboelectric system, comprising elastic resin and polydimethylsiloxane (PDMS), both of which exhibit superhydrophobic and self-cleaning properties. The hierarchical micro/nanostructures of the lotus leaf have been replicated in these layers. The utilization of bioinspired micro/nanostructures resulted in a twofold enhancement of the output of the BSRW-TENG. Additionally, these structures imparted exceptional resistance to contamination and humidity, thereby conferring sweat-resistance to the BSRW-TENG. Moreover, the BSRW-TENG exhibited remarkable resistance to humidity, exhibiting a mere 11% reduction in output as the relative humidity escalated from 10% to 80%. In contrast, the flat-TENG experienced a 54% decline. The capacity of the material to resist sweat was additionally confirmed in severe and challenging circumstances, such as complete surface pollution and exposure to highly humid water spraying. The BSRW-TENG exhibits significant potential for cost-effective monitoring of personal exercise and analysis of athletes’ training. The study presents a newly developed technique for generating gaps in situ in a manner that is environmentally friendly. This method involves the vaporization of distilled water that has been soaked and is utilized in the fabrication of a triboelectric nanogenerator that does not require a spacer. This is illustrated in Figure 5d. The present study demonstrates that the NSTENG exhibits more homogeneous stress/strain patterns and experiences greater displacement in comparison to the conventional TENG with a spacer under identical pressure conditions. Furthermore, the NSTENG effectively eliminates any hindrance in detecting minor movements in the vicinity of the spacer. The distinctive manufacturing process additionally ensures biological security and circumvents in vivo air pollution. The wireless mobile system that is currently in place, which is founded on the NSTENG, is capable of accurately detecting complete pulse waveforms and presenting them on the screen of a mobile device without delay. Through attachment to the cardiac organ of a rat, the device is capable of monitoring typical cardiac motion and the resultant heart rate measurement exhibits a precision of up to 99.73%. In addition, the NSTENG has the capability to oversee anomalous cardiac motion and identify minute cardiac movements that are not recorded by the electrocardiogram. The present study presents a novel approach that facilitates the advancement of biosafe and innovative TENGs with new structures and provides fresh perspectives on the development of sensors that can be worn or implanted. The depiction in Figure 6a illustrates the utilization of an in-shoe sensor pad (ISSP) affixed to the vamp lining, which operates on the principles of a triboelectric nanogenerator (TENG) to facilitate the monitoring of stress distribution on the dorsal surface of the foot in real time. The ISSP is equipped with air-capsule triboelectric nanogenerators (AC-TENGs), which comprise activated carbon/polyurethane (AC/PU) and microsphere array electrodes. The multifunctional Insole Sensing System Platform (ISSP) has the capability to perform various standard functions of traditional smart footwear, such as step counting and interaction between humans and machines. Furthermore, it has the potential to unveil specific details, such as the footwear’s level of fitness, the concentration of stress on the toes, and the degree of comfort experienced during motion. The system incorporates signal processing and data transmission modules that are equipped with a hybrid power supply featuring wireless power transfer technology. Additionally, the system enables real-time monitoring of foot-related information through a mobile device. Therefore, the present study proposes an ISSP as a viable method to investigate the motion of feet and the level of comfort provided by shoes during extended periods of use. This approach has the potential to inform athlete training and the development of tailored shoe designs. The findings presented in Figure 6b demonstrate the utilization of a nanostructured silk protein and silver nanowires (AgNWs) that are embedded in the silk nanostructure. This approach results in the development of a triboelectric nanogenerator (TENG) and strain sensor that is both efficient and flexible, while also being transparent and compatible with skin and textile materials. The resulting device is capable of harvesting biomechanical energy and sensing motion, making it a promising candidate for various applications. The bio-TENG’s optical transparency renders it a viable option for utilization as a touch sensor on electronic devices. 

The integration of a strain sensor and a bio-TENG into a singular silk chip, which is subsequently affixed to both skin and fabrics, enables the simultaneous monitoring of strain and harvesting of biomechanical energy. The protein-based energy skin offers several benefits, such as affordability, simplicity of production, compatibility with living organisms, pliability, and translucency. These advantages make it a suitable material for various applications, including a smooth interface between humans and machines, touch-sensitive devices, and wearable bioelectronic devices. The study showcases an inventive implantable triboelectric nanogenerator (iTENG) designed for the purpose of in vivo biomechanical energy harvesting, as depicted in Figure 6c. The biomechanical energy conversion devices’ in vivo output performance was compared to the improved output voltage and corresponding current, which were achieved by utilizing the heartbeat of adult swine. The results showed a significant increase of 3.5 times in voltage and 25 times in current. Furthermore, the iTENG was subjected to in vivo assessment for a duration exceeding 72 h of implantation, wherein it exhibited uninterrupted generation of electrical energy in the live subject. A self-sustaining wireless transmission system was constructed for the purpose of real-time wireless cardiac monitoring, owing to its exceptional in vivo efficacy. Due to its exceptional in vivo performance and durability, iTENG has the potential to be utilized not only for powering implantable medical devices, but also for the development of a self-sustaining, wireless healthcare monitoring system. The flexible single-electrode triboelectric nanogenerator (S-TENG) illustrated in Figure 6d is constructed using a multi-walled carbon nanotube (MWCNT)/polydimethylsiloxane (PDMS) film. The S-TENG exhibits several benefits such as exceptional flexibility, remarkable hydrophobicity, negligible weight, economical cost, and superior output efficiency. The S-TENG has the capability to extract energy from various sources such as falling water droplets, continuous water flow, wind, and percussion sounds. This feature showcases its vast potential for utilization in the realm of flexible wearables.

### 3.3. Integration TENG with Other Technologies for Motion Sensing

**Figure 7:**
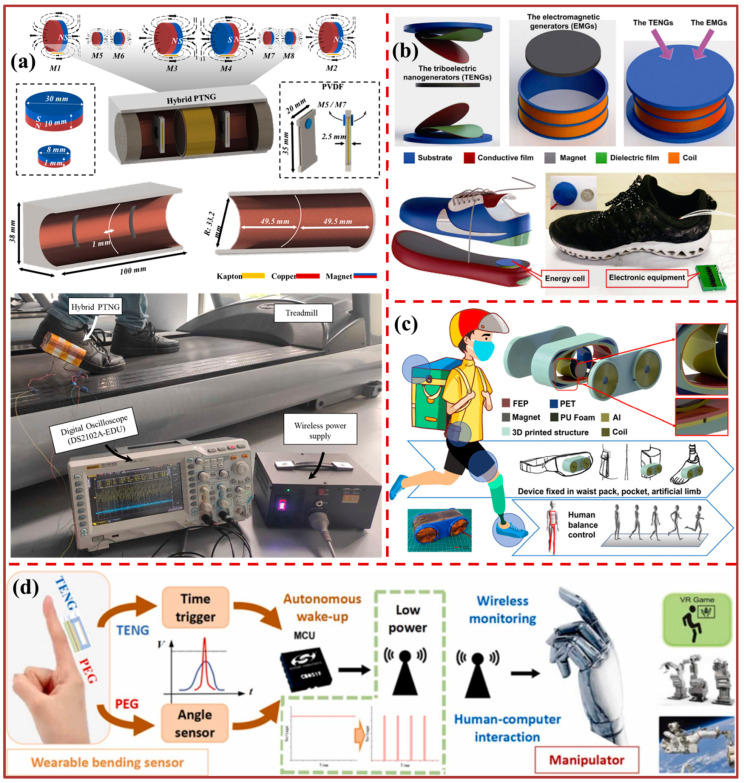
Integration of TENG with other technologies for motion sensing: (**a**) Application of hybrid PTNG for motion sensing [3]. (**b**) Self-powered versatile shoes based on hybrid nanogenerators [103]. (**c**) Schematic diagram of the HETNG designed for scavenging biomechanical energy in human balance control [104]. (**d**) Self-powered wearable bending wireless sensing with autonomous wake-up [105].

**Figure 8:**
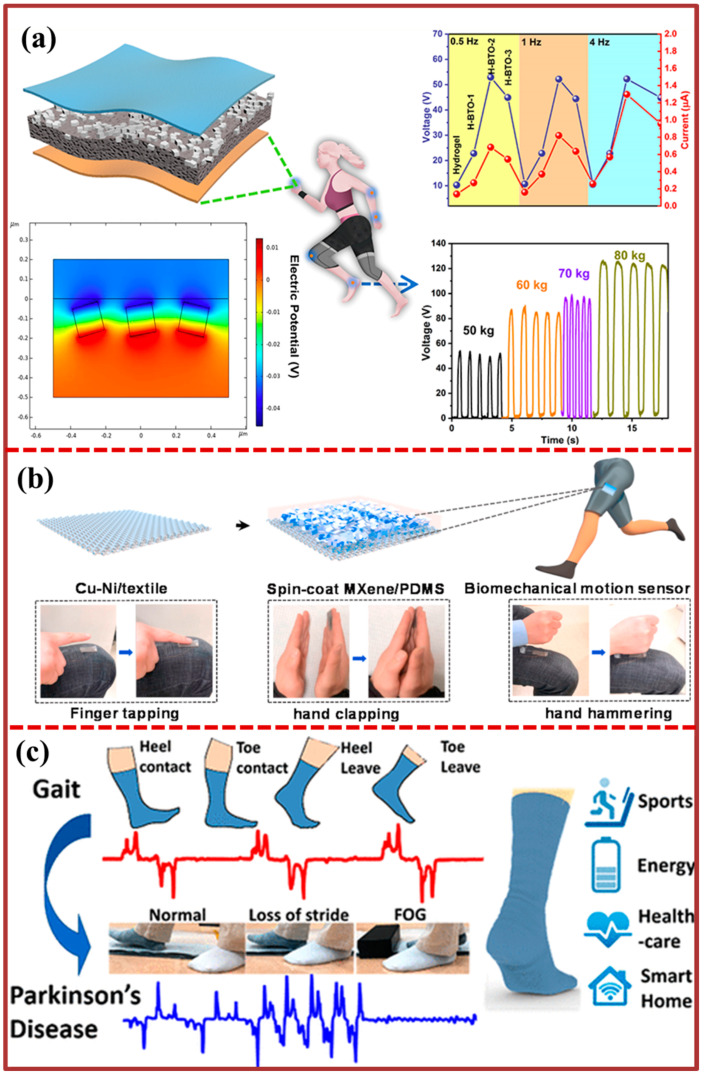
Integration TENG with other technologies for motion sensing: (**a**) Detection of different human motions and flexural measurement using the flexible multifunctional PAM/BTO composite film-based sensor [106]. (**b**) Flexible single-electrode triboelectric nanogenerators with MXene/PDMS composite film for biomechanical motion sensors [88]. (**c**) Design principal and application of PEDOT for Parkinson’s disease [107].

Figure 7 and Figure 8 show the amalgamation of triboelectric nanogenerators with other technologies to facilitate motion sensing. A novel hybrid nanogenerator combining piezoelectric and triboelectric effects, denoted as PTNG, has been developed and presented in Figure 7a for the purpose of energy generation and monitoring. The PTNG employs magnetic force to enact the counteracting force during the sliding mode between the Kapton and copper/aluminum layers in the triboelectric component, as well as in the polyvinylidene fluoride strips. 

The present study details the development of a self-powered walking sensing system that utilizes the PTNG to analyze human behavior while walking on a treadmill. This methodology potentially offers a novel avenue for the development of high-efficiency and controllable energy harvesting devices that can leverage human movements to generate enhanced power output. The mini-sized hybrid nanogenerator depicted in Figure 7b has the potential to function as an energy cell when integrated into footwear. The successful demonstration of typical outdoor applications, such as driving with a Global Positioning System (GPS) device, charging a Li-ion battery, and a cell phone, indicates the potential application of this technology in smart wearable electronics and future military attire. Figure 7c shows the development of a hybridized electromagnetic–triboelectric nanogenerator (HETNG) with the aim of harnessing biomechanical energy generated during human balance control processes, while also facilitating effective monitoring capabilities. The high energy trajectory net generator (HETNG) is comprised of a pendulum structure that exhibits symmetry and a cylindrical magnet that undergoes rolling motion within the structure. The four coils are partitioned into two distinct clusters, each of which constitutes a separate electromagnetic generator (EMG). In addition, a freestanding mode triboelectric nanogenerator (TENG) is composed of two spatial electrodes affixed to the inner wall. In addition to the human balance control mechanisms involved in walking, the HETNG has the ability to extract biomechanical energy from various locations on the trunk. Additionally, the high electromyography (EMG) technique for noninvasive gait analysis (HETNG) has been simulated in the context of artificial limb implementation. Specifically, the HETNG has been applied to the thigh and foot positions to monitor the actions of squatting, standing up, and lifting the leg up and down. The implementation of a health and environmental tracker with notification gateway (HETNG) on the walking aid of elderly individuals who exhibit slow gait and require assistance can facilitate the recording of forward movements and unforeseen falls. This feature can prove to be beneficial in emergency situations in which prompt assistance is required. The present study demonstrates the capacity of biomechanical energy-based hybrid energy transfer nanogenerators (HETNG) in energizing portable electronic devices and tracking human movements. Additionally, it highlights the noteworthy implications for individuals with limited mobility or disabilities. A proposed design for a self-powered wearable sensor is depicted in Figure 7d, featuring a flexible hybrid nanogenerator with a bridge structure. The process of extracting energy from the motion of a bent finger involves the integration of the contact separation mode for the triboelectric nanogenerator (TENG) with the bending d31 mode for the piezoelectric generator (PEG). These designs propose an autonomous wireless sensing method utilizing a hybrid generator in wearable bending to underscore its significance. In contrast to continuous data recording and transmission, the utilization of triboelectric nanogenerator (TENG) signals as a trigger mechanism enables the recording of piezoelectric generator (PEG) voltage amplitude as angle sensing data, which can then be wirelessly transmitted. Autonomous wake-up technology is anticipated to decrease the computational load and power usage of wireless sensing, while also enabling more opportunities for wearable wireless monitoring and human–computer interaction by providing precise and succinct sensing data. The experiment, involving wireless manipulator interaction and utilizing a hybrid nanogenerator as a wearable bending sensor, has demonstrated the viability of this approach. This scheme holds significant potential for application in the field of wearable self-powered sensors, including virtual reality and robot control. The study presents Figure 8a, which exhibits a TENG that is characterized by flexibility, stretchability, and high transparency. This TENG is based on an unsymmetrical composite film composed of polyacrylamide (PAM) hydrogel and BaTiO_3_ nanocubes (BTO NCs, BTO). The performance of the TENG can be customized by modifying the quantity and spatial arrangement of BTO. The hydrogel electrode exhibits a remarkable capacity to withstand stretching, with a capacity exceeding eight-fold. The enhancement of the output of the fabricated triboelectric nanogenerators (TENGs) to function as high-sensitivity pressure sensors capable of distinguishing a range of forces (0.25–6 N) at low frequencies was achieved by modifying the content and distribution location of BTO in the unsymmetrical hydrogel film. This paper provides a detailed discussion on the mechanism behind the improved output performance observed in the PAM/BTO composite hydrogel-based triboelectric nanogenerator. The optimized TENG and piezoresistive sensors have been integrated to detect the motions of human bodies, pressure, and curvature with high sensitivity. This has been achieved by combining piezoresistive, piezoelectric, and triboelectric effects, resulting in multimodal biomechanical sensors. The flexible single-electrode triboelectric nanogenerator, which is composed of MXene/polydimethylsiloxane film, is depicted in Figure 8b. The present study systematically investigates the impact of (MXene) content (measured in weight percentage) and compressive force on the output performance of the devices that were prepared. 

The elevated electrical yield facilitates the direct powering of a series-connected set of 80 green-light-emitting diodes, without the need for supplementary power sources. The textile-based composite material serves a dual purpose as both a triboelectric nanogenerator and a human motion sensor, capable of detecting a range of human movements including finger tapping, hand clapping, and hand hammering. The utilization of lightweight smart textiles presents a range of potential opportunities for multifunctional power sources, thereby exhibiting promising applications in the domain of self-powered wearable electronics. Figure 8c shows a self-sustaining and autonomously operational sock, denoted as S2-sock, which has been developed to encompass a spectrum of functionalities including energy acquisition and the detection of an array of physiological indicators, such as gait dynamics, tactile force, and perspiration levels. This innovation is achieved through the synergistic integration of a fabric-based triboelectric nanogenerator (TENG) coated with poly(3,4-ethylenedioxythiophene) polystyrenesulfonate (PEDOT:PSS) and piezoelectric chips constructed from lead zirconate titanate (PZT). The investigation further explores the ramifications of ambient humidity, temperature fluctuations, and load variations, demonstrating that the S2-sock effectively realizes gait analysis and movement monitoring for intelligent residential applications. Building upon the foundation of sensor fusion, the data streams originating from TENG and PZT sensors during physical activities are seamlessly amalgamated, facilitating rapid assessment of perspiration levels. Leveraging the amalgamated capabilities of the hybrid S2-sock, a broader array of functions, encompassing foot-based energy harvesting and comprehensive monitoring of diverse physiological cues pertinent to healthcare and smart home contexts, can be accomplished.

## 4. Challenges in Skin-Contact Triboelectric Nanogenerator Applications

As a popular choice in wearable devices, skin-contact TENG offers an effective solution for energy-harvesting as well as motion-sensing applications [108]. The mechanical movements of the body are used to generate electrical charges via the triboelectric effect in the skin-contact TENG. Although skin-contact TENG technology shows great potential, it faces several significant challenges that must be addressed to ensure its practicality and effectiveness. This section will discuss these challenges, particularly the sensitivity of skin-contact TENGs to environmental factors, biocompatibility and safety concerns, and durability and reliability issues [108,109,110]. One of the primary challenges faced by skin-contact TENGs is their sensitivity to environmental factors. Skin-contact TENGs are highly sensitive to external factors, such as temperature, humidity, and mechanical vibrations, which can significantly impact their performance. Changes in these factors can also influence the amount of electrical energy generated by the TENG [111,112,113,114,115]. Therefore, it becomes crucial to understand the environmental factors that can affect the performance of skin-contact TENGs by conducting extensive research on the TENGs against various environmental factors. The optimization of materials, structure, and design can also be undertaken according to environmental factors by taking into account the factors affecting the performance of the TENG [116]. Another major challenge associated with skin-contact TENG technology is biocompatibility and safety concerns [117]. The materials used in skin-contact TENGs must be biocompatible and safe for use on human skin. It is imperative to ensure that the materials used do not cause skin irritation, rashes, or other adverse health effects [118]. The biocompatibility of the TENGs must be tested rigorously and repeatedly to ensure their safety for human use. Biocompatibility testing of these materials, therefore, becomes a necessity to establish their safety and efficacy. Materials used in TENGs need to be verified in terms of their compatibility with humans to ensure their use in practical applications [119,120,121,122,123,124,125]. Additionally, durability and reliability issues are major obstacles faced by skin-contact TENGs. Repeated movements, exposure to environmental factors, and regular wear and tear can cause damage to the TENGs, leading to reliability issues. Durability and reliability should be the primary concerns during production and design, and the integrity of the materials used should be considered for longer usage. The use of high-performance materials to withstand movement, wear and tear, and the ability to sustain over an extended period should be a top priority during the design and development process [126,127]. Furthermore, biomaterial-based TENGs, exemplified by silk protein, offer the advantage of inherent biocompatibility and potential integration with biological systems, making them suitable for wearable devices in healthcare applications. However, their mechanical and electrical properties might limit energy generation efficiency. Conversely, conventional polymeric TENGs like PDMS provide robust mechanical properties and compatibility with various fabrication processes, allowing for efficient energy conversion. Yet they might lack the inherent biocompatibility of biomaterials. Balancing biocompatibility and energy harvesting efficiency presents a challenge for biomaterial-based TENGs, while conventional polymeric TENGs could require additional measures for safe skin contact.

## 5. Perspectives and Future Directions

Skin-contact TENG devices can harness the energy generated by human body movements to generate electricity. This study provides a comprehensive analysis of the perspectives and future directions of skin-contact TENG technology, covering advancements in materials and design, potential for commercialization, and impact on energy and healthcare industries [127]. Advancements in materials and design are key for future research in skin-contact TENG technologies. These can lead to improved performance, increased biocompatibility, and enhanced durability of the TENGs. New materials have the potential to increase the effectiveness of TENGs, such as graphene or silver nanowires [128]. Flexible, lightweight, and biocompatible materials are essential for the development of skin-contact TENGs. Additionally, the ability to integrate nanomaterials with enhanced electrostatic properties into the surface of TENGs could increase energy conversion efficiency [129,130,131]. Improved designs that decrease sensitivity to environmental factors, such as humidity or temperature, increase the practicality of skin-contact TENGs. Skin-contact TENG technology has high potential for commercialization, particularly for energy-harvesting applications. As demand for wearables in healthcare, entertainment, and fitness continues to rise worldwide, they offer a practical solution to the replacement of traditional energy sources, such as batteries. Energy-efficient and portable TENG technology can potentially reduce waste and energy consumption, revolutionizing the energy industry. Moreover, skin-contact TENGs could be applied in various commercial products, including smart fabrics, self-powered sensors, and mobile devices. Some industries and start-ups have incorporated TENG technology into their products, demonstrating its commercial feasibility [132]. With continued investment in energy and healthcare industries, skin-contact TENG technology could offer practical solutions to various challenges in energy harvesting and healthcare monitoring. The impact of skin-contact TENGs on the energy and healthcare industries could be significant. TENG technology improves sustainability and promotes innovation in energy harvesting [133]. Energy-efficient TENGs have the potential to decrease the demand for traditional energy sources, resulting in less waste and energy consumption. The healthcare industry can also benefit from skin-contact TENG technology. TENGs can be used for remote healthcare monitoring of body movements, such as monitoring heart rate, blood pressure, and respiration rate. Healthcare professionals can provide personalized, more effective treatment for their patients because of the information provided by TENGs [134]. Moreover, the ability to integrate skin-contact TENG technology with other technologies has the potential for various applications, such as RFID tags, wireless sensors, or internet of things (IoT) devices. Skin-contact TENG technology could lead to significant progress and optimization within the healthcare industry, wearable technology, and beyond [93]. To enhance the sensitivity of skin-contact triboelectric nanogenerator (TENG) devices, several aspects could be explored. Firstly, the optimization of triboelectric material selection, including advanced polymers and composite materials, could amplify charge generation through improved triboelectric properties. Incorporating micro/nanostructuring on the material surfaces might increase contact area and enhance charge separation. Furthermore, the exploration of innovative electrode designs and layouts could facilitate efficient charge collection. Integrating optimized mechanical structures that enhance the stretching and compression of TENG components during motion could maximize energy conversion. Developing advanced signal conditioning and amplification techniques, along with noise reduction methods, could enhance motion sensing sensitivity. Exploring adaptable contact mechanisms and real-time tuning to match varying conditions during motion could improve charge transfer efficiency. Leveraging machine learning algorithms for signal analysis could enable precise motion detection. By addressing these aspects, the sensitivity of skin-contact TENG devices could be significantly increased, leading to improved energy-harvesting and motion-sensing capabilities [18,41,135,136].

## 6. Conclusions

In conclusion, this review paper presents a comprehensive evaluation of skin-contact TENG technology, covering everything from the basic principles of energy harvesting to the potential impact of the technology on the energy and healthcare industries. The paper highlights the importance of energy harvesting and motion sensing, and how skin-contact TENG technology has emerged as a promising solution in this field. The paper explains the principles of skin-contact TENG, focusing on key concepts such as the triboelectric effect and materials used for energy harvesting. Moreover, the paper discusses the working mechanism of skin-contact TENG and explores the potential applications of this technology in energy harvesting for wearable devices and motion sensing for healthcare monitoring. Additionally, the integration of skin-contact TENG technology with other technologies is discussed, highlighting the versatility of the technology. However, the paper judiciously accentuates a constellation of formidable challenges intrinsic to the skin-contact TENG landscape, each serving as a critical impetus for meticulous refinement. The discerning scrutiny unveils a triad of paramount challenges that intricately interweave environmental sensitivity, biocompatibility, and safety considerations, alongside the paramount domains of durability and reliability. The granular exploration of these challenges underscores their pivotal role as linchpins in the realization of skin-contact TENG’s practical utility within energy harvesting and motion sensing contexts. The subsequent Perspectives and Future Directions segment, serving as a beacon for researchers and stakeholders, plunges into a panoramic vista of recent strides in materials and design innovations. It expounds upon the central significance conferred upon flexible, lightweight, and biocompatible materials, emphasizing their profound potential to serve as linchpins for the holistic augmentation of skin-contact TENG’s performance metrics. Notably, the discussion magnanimously transcends the theoretical expanse to probe the fringes of commercialization, resonating palpably within the energy and healthcare sectors. The nuanced contemplation of skin-contact TENG’s convergence with commercial paradigms crystallizes into a powerful testament to its transformative potential as an agent of change. In a final crescendo, the paper illuminates the far-reaching ripples that skin-contact TENG technology may catalyze within the energy and healthcare spheres. With emphatic articulation, it underscores the urgency for sustained research and development, epitomizing the trajectory towards unlocking the technology’s latent promise. In summation, the paper unfurls a carefully woven tapestry that blends challenges, advancements, commercial pathways, and societal impact, solidifying its position as a lodestar for propelling skin-contact TENG towards a future steeped in transformative significance. Overall, this review paper provides a comprehensive overview of skin-contact TENG technology, covering its principles, challenges, and potential applications. The paper highlights the significant potential and versatility of skin-contact TENGs in energy harvesting and motion sensing and identifies the technological limitations that must be addressed for practical applications. The Perspectives and Future Directions section provides valuable insights for researchers and industry professionals interested in the potential of skin-contact TENG technology for energy harvesting and motion sensing.

## Data Availability

The data presented in this study are available on request from the corresponding author.
